# Supplementary material for: Crl activates transcription by stabilizing active conformation of the master stress transcription initiation factor
Source: eLife. 2019 Dec 17;8:e50928. doi: 10.7554/eLife.50928 (PMC6917491; doi:10.7554/eLife.50928)
Supplement: Supplementary file 1. [file elife-50928-supp1.docx]

|  | ***E. coli* Crl-TAC** |
| --- | --- |
| **Data collection** |  |
| Number of grids used | 1 |
| Grid type | Holey carbon |
| Microscope/detector | Titan Krios/Gatan K2 |
| Voltage (kV) | 300 kV |
| Dose rate (e^-^/s) | 6.7 |
| Pixel size (Å/pix) | 0.507 |
| Total dose (e^-^/ Å^2^) | 53.6 |
| Total exposure time (s) | 8 |
| Number of frames/movie | 32 |
| Defocus range (μm) | 2.0-2.5 |
| Number of micrographs | 3,290 |
| Particles used for final map | 184,208 |
| **Model composition** |  |
| Non-hydrogen atoms | 26527 |
| Protein residues | 3499 |
| Nucleotide | 97 |
| Ligands (Zn2+/Mg2+) | 2/1 |
| **Refinement** |  |
| Resolution (Å) | 3.80 |
| Map sharpening B factor (Å^2^) | -142.985 |
| Average B factor (Å^2^) |  |
| Protein | 35.98 |
| Nucleotide | 90.89 |
| Ligand | 65.89 |
| Clash score | 4.72 |
| RMS deviations |  |
| Bond lengths (Å) | 0.009 |
| Bond angles (°) | 1.097 |
| Ramachandran plot |  |
| Favored (%) | 85.7 |
| Allowed (%) | 14.27 |
| Outliers (%) | 0.03 |
