# Supplementary material for: Crl activates transcription by stabilizing active conformation of the master stress transcription initiation factor
Source: eLife. 2019 Dec 17;8:e50928. doi: 10.7554/eLife.50928 (PMC6917491; doi:10.7554/eLife.50928)
Supplement: Supplementary file 2. [file elife-50928-supp2.docx]

|  | ***K_D_* (nM)** | **Ratio (*K_D, Crl_* / *K_D_*_,_ *_none_*)** |
| --- | --- | --- |
| None | 40 ± 4.0 | 1.0 |
| Crl (WT) | 9.0 ± 1.0 | 0.2 |
| Crl (ΔN-tail) | 15 ± 1.0 | 0.4 |
| Crl (ΔR-loop) | 30 ± 4.0 | 0.8 |
| Crl (ΔN-tail /ΔR-loop) | 35 ± 2.0 | 0.9 |
